# Compression of the median nerve in the proximal forearm by a giant lipoma: A case report

**DOI:** 10.1186/1749-7221-3-17

**Published:** 2008-06-10

**Authors:** Sebastian E Valbuena, Greg A O'Toole, Eric Roulot

**Affiliations:** 1 Deparment of Orthopeadic Surgery & Traumatology, Hospital Interzonal El Cruce, Alta complejidad en red. Florencio Varela, Buenos Aires, Argentina; 2 Institut de la Main, Clinique Jouvenet, Paris, France

## Abstract

**Background:**

Compression of the median nerve by a tumour in the elbow and forearm region is rare. We present a case of neuropathy of the median nerve secondary to compression by giant lipoma in the proximal forearm.

**Case presentation:**

A 46-year-old man presented with a six month history of gradually worsening numbness and paresthesia on the palmar aspect of the left thumb and thenar eminence. Clinical examination reveals a hypoaesthesia in the median nerve area of the left index and thumb compared to the contralateral side. Electromyography showed prolonged sensory latency in the distribution of the median nerve corresponding to compression in the region of the pronator teres (pronator syndrome). Radiological investigations were initially reported as normal. Conservative treatment for one month did not result in any improvement. Surgical exploration was performed and a large intermuscular lipoma enveloped the median nerve was found. A complete excision of the tumour was performed. Postoperative revaluation the X-ray of the elbow was seen to demonstrate a well-circumscribed mass in the anterior aspect of the proximal forearm. At follow-up, 14 months after surgery, the patient noted complete return of the sensation and resolution of the paresthesia.

**Conclusion:**

In case of atypical findings or non frequent localization of nerve compression, clinically interpreted as an idiopathic compression, it is recommended to make a pre-operative complementary Ultrasound or MRI study.

## Background

Compression of the median nerve in the elbow and proximal forearm region is much less frequent than within the carpal tunnel [1]. Proximal compression is most commonly the result of anatomic variations with the supracondylar process and Struthers ligament [2], the lacertus fibrosus (bicipital aponeurosis), the pronator teres muscle and the arch of the flexor superficialis most commonly implicated [3]. With less frequency, anomalous anatomic structures are implicated in compression of the median nerve, these being most commonly, the accessory head of the flexor pollicis longus (Ganzer's muscle) [4], and a persistent median artery [5].

Rarer causes of extrinsic compression of the median nerve such as chronic compartment syndrome [6], partial rupture of the distal biceps insertion [7], and synovial osteochondromatosis at the elbow [8] have also been reported.

The compressive neuropathy of the median nerve secondary to lipoma is not frequent, and has been described principally in the wrist and the hand [9-12]. We present a case of compression of the median nerve in the proximal forearm by a giant lipoma.

## Case presentation

A 46-year-old man presented with a six month history of gradually worsening numbness and paresthesia on the palmar aspect of the left thumb and thenar eminence.

Static two point discrimination in the median nerve distribution of the index and thumb showed a hypoaesthesia compared to the contralateral side. Tinel's sign of the wrist and forearm and Phalen's sign were negative. Active forearm pronation against resistance in slight flexion, resisted active forearm supination and resisted active index and middle finger flexion did not elicit pain. Grip strength was equal bilaterally. No masses were detectable on examination of the hand, wrist and forearm. No history of vaccination, viral infection or medication within the previous year was offered.

**Figure 1:**
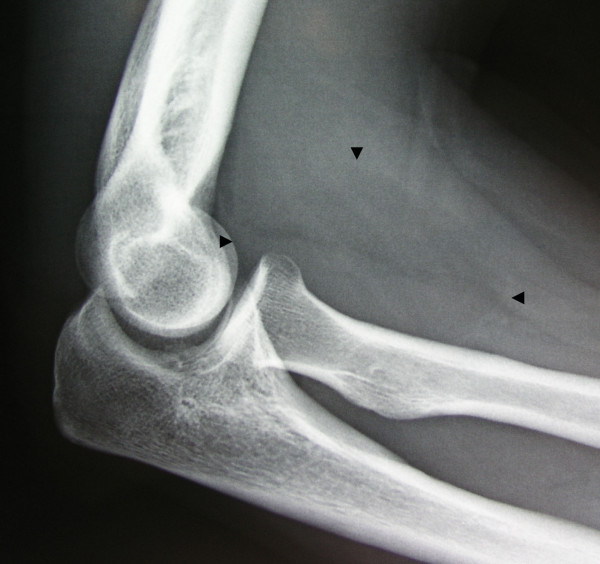
A lateral elbow X-ray subtly demonstrates a well circumscribed mass in the anterior proximal forearm (arrows).

X-rays of the cervical vertebrae, elbow, forearm and hand were initially reported as normal. However, on postoperative revaluation the X-ray of the elbow was seen to demonstrate a well-circumscribed mass in the anterior aspect of the proximal forearm (Figure 1). Electromyography showed prolonged sensory latency in the distribution of the median nerve corresponding to compression in the region of the pronator teres (pronator syndrome).

**Figure 2:**
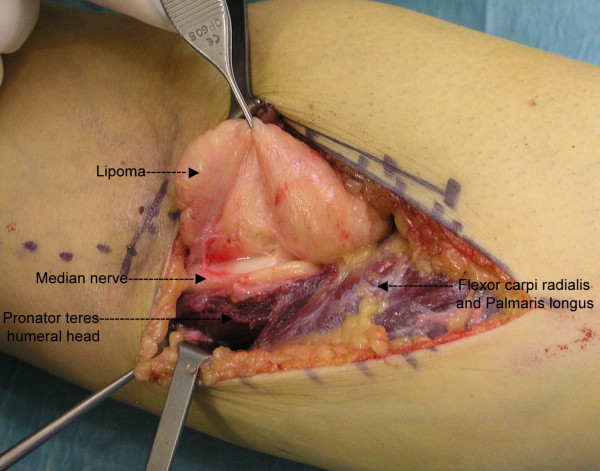
Intraoperative photo showing the median nerve within the intermuscular lipoma.

**Figure 3:**
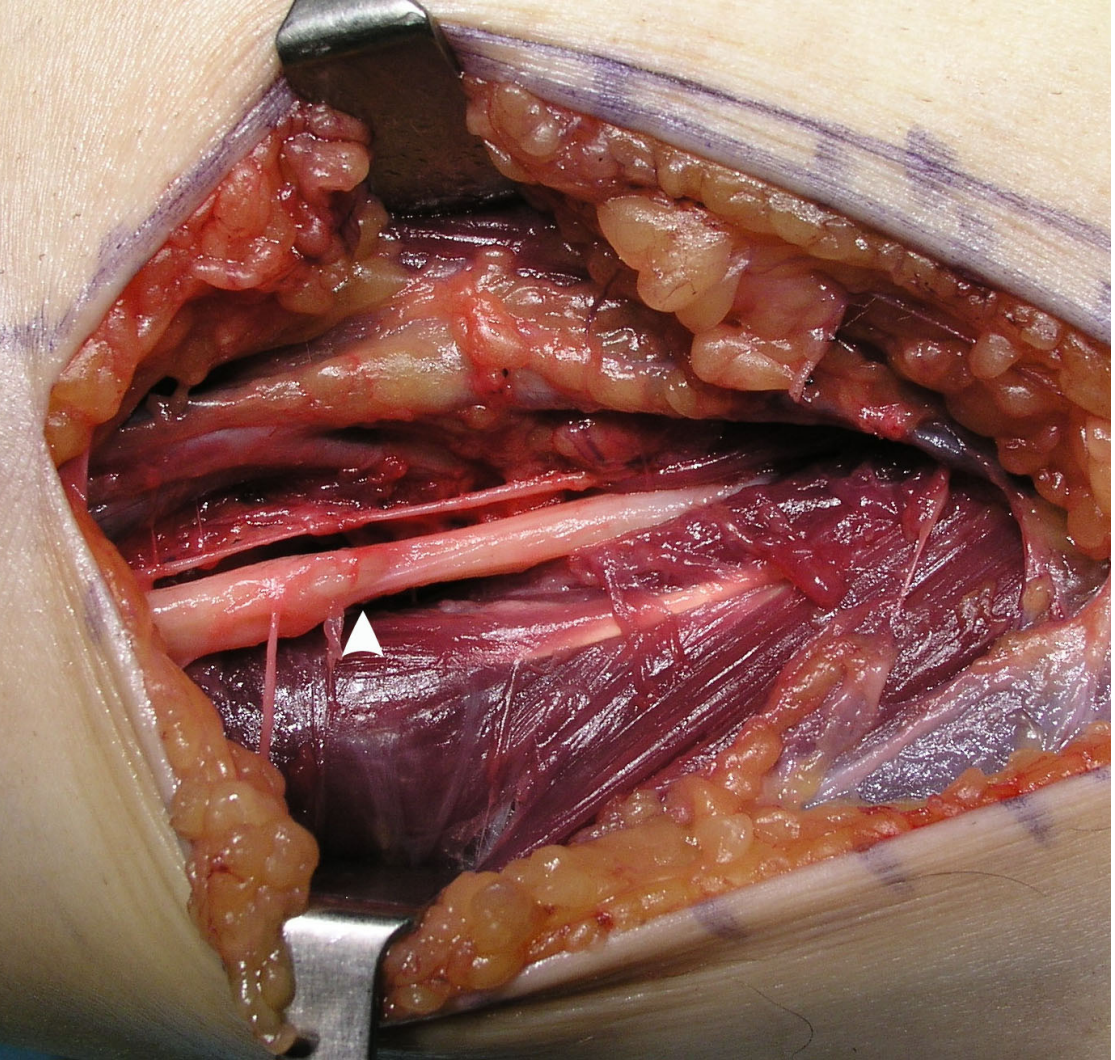
Surgical area after lipoma excision: the arrow shows the hourglass deformation of the nerve at the level of the compression.

**Figure 4:**
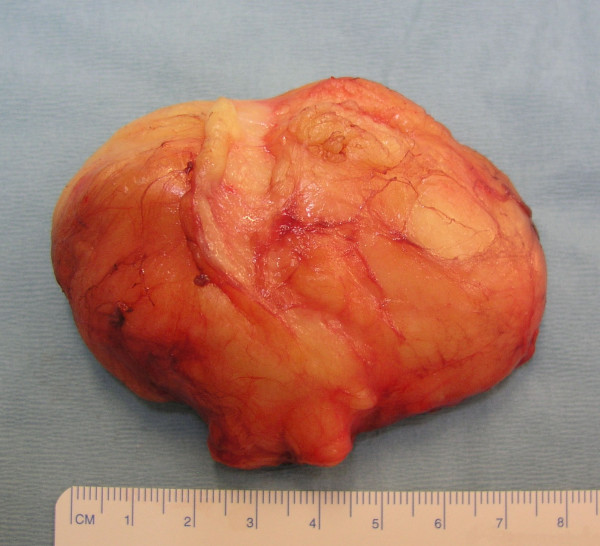
The giant lipoma was excised completely and measured 8 cm × 6 cm × 3 cm.

Conservative treatment (anti-inflammatory medication and a diurnal long-arm splint) for one month did not result in any improvement. Surgical exploration was therefore performed under regional anesthesia and hemostatic tourniquet. The surgical incision began just medial to the biceps tendon and distal to the elbow flexion crease and continued to the mid-forearm between the flexor and extensor muscle masses. The medial antebrachial cutaneous nerve was identified and retracted. The pronator mass and the biceps tendon were identified. An intermuscular mass of adipose tissue was identified just lateral to the superficial head of the pronator teres, the dissection was not difficult but the median nerve was enveloped by the tumour (Figure 2). Microsurgical techniques were used to allow an extracapsular and non-traumatic dissection. A complete excision of the tumour of 8 cm × 6 cm × 3 cm was performed (Figure 4). The median nerve had an hourglass deformity as a result- of its compression (Figure 3). Histopathological examination of the tissue removed at surgery confirmed the presence of well-differentiated mature fat cells (lipoma). There were no neural or neoplastic features.

At follow-up, 14 months after surgery, the patient noted complete return of the sensation and resolution of the paresthesia.

## Discussion

Lipomas are benign tumours originating from adipose cells occurring in subcutaneous tissues, intermuscular, intramuscular or paraosteal localizations [9]. Lipomas of more than 5 cm diameter (Giant lipoma) are infrequent in the upper limb [10]. In this anatomic location, masses are generally symptomatic at a smaller size.

Nerve compression by a lipoma is uncommon [9,13] but subfascial lipomas are deep tumours and can be a cause of nerve compression [14].

Review of English literature on extrinsic nerve compression by lipoma in the upper limb reveals several reports of compression of the radial nerve (especially the posterior interosseous nerve due to the anatomic relation with the neck of the radius) [8,15-19], and a few cases of ulnar nerve compression in the forearm [11] and the Guyon's canal [20-22]. The involvement of the median nerve was also reported in the brachial plexus area's [23] and particularly in the wrist or the palmar region's [9-12,24-27]. Only one case documented an extrinsic compression of the medial nerve in the proximal forearm by a giant lipoma resulting in an anterior interosseous syndrome [28]. To our knowledge, the case presented is the second reported case of compression of the median nerve in the proximal forearm by a giant lipoma.

Cribb et al. [10] documented a series of 10 giant lipomatous tumours (7 lipomas, one neural fibrolipoma and two well differentiated lipoma-like liposarcomas), five cases were in the hand and five cases in the forearm, with signs of median nerve compression in two cases, one in the hand with the location of compression in the second case being unclear. However, in all cases neurovascular structures required mobilisation in order to excise the tumour.

Cribb et al. [10] stressed the importance of a multidisciplinary approach to investigations of giant soft tissue tumours and suggested that an MRI should be routine. In cases where an MRI does not clearly demonstrate a lipoma or in those patients who could not tolerate the investigation, they go on to suggest that a biopsy be performed.

Johnson et al. [29] demonstrated that soft tissue masses of greater than five cm in diameter should be considerate malignant unless proven otherwise.

Marginal resection with conservation of the neurovascular structures is the procedure of choice for lipomas, and a more aggressive surgery is required in case of malign tumour. In our case the diagnosis of tumour compression, despite of the size of the lipoma, was made intra-operatively as we had considered the atypical clinical findings to be the result of idiopathic compression of the median nerve in the elbow region.

Ultrasound is an excellent diagnostic study, especially for deeply sited masses and can be used such cases. MRI however, provides more information of tumour type and of anatomic relations and is therefore preferable for diagnostic precision and pre-operative planning.

## Conclusion

Extrinsic median nerve compression by a tumour is rare. However, in case of atypical findings or non frequent localization of nerve compression, clinically interpreted as an idiopathic compression, it is recommended to make a pre-operative complementary Ultrasound or MRI study.

## Competing interests

The authors declare that they have no competing interests.

## Authors' contributions

SEV, GAO, and ER conceived the case report and interpreted the data, SEV performed all pertinent literature review on the subject and drafted the manuscript, ER performed the patient's surgery and collected the clinical data, SEV assisted to ER in the surgery, GAO helped to draft the manuscript.

All authors read and approved the final manuscrit.
